# A case involving a submucosal bulge in the duodenal bulb was ultimately confirmed to be a residual gallbladder

**DOI:** 10.1055/a-2776-5497

**Published:** 2026-01-30

**Authors:** Weijia Zhu, Heng Zhang, Xi Chen, Aihua Qian

**Affiliations:** 1 Department of Gastroenterology, Wuxi Branch of Ruijin Hospital, Shanghai Jiao Tong University School of Medicine, Wuxi, China; 2 66281 Department of Pathology, Shanghai Jiao Tong University Medical School Affiliated Ruijin Hospital, Shanghai, China; 3 66281 Department of Gastroenterology, Shanghai Jiao Tong University Medical School Affiliated Ruijin Hospital, Shanghai, China

**Fig. 1:**
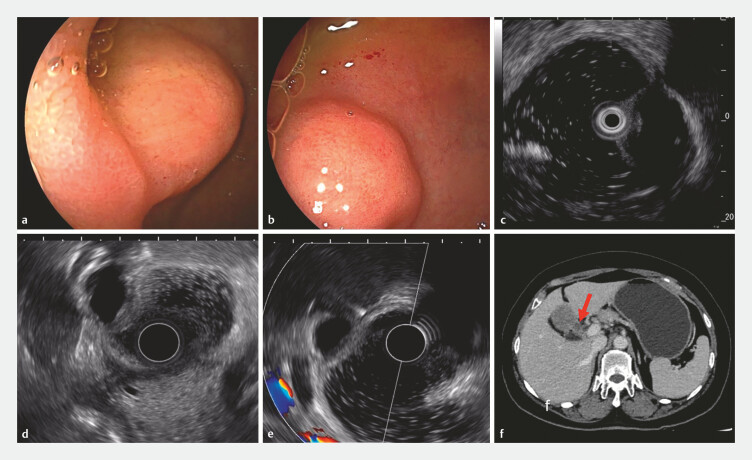
Images of submucosal bulge ESD preoperative evaluation: **a, b** white light endoscopy; **c** MPS; **d, e** EUS; **f** enhanced CT for preoperative staging of gastric cancer. CT, computed tomography; ESD, endoscopic submucosal dissection; EUS, endoscopic ultrasonography; MPS, mucosal protrusion sign.

A 66-year-old woman underwent gastroscopy due to epigastric discomfort. The procedure revealed an 18 × 10 mm submucosal bulge located in the duodenal bulb near the anterior pyloric wall ( Fig. 1 **a, b** ). The mucosal protrusion sign suggests an origin in the intrinsic muscularis propria and is characterized by hypoechoic appearance ( Fig. 1 **c** ). Subsequent endoscopic ultrasonography corroborated these findings, and no blood flow signals were detected ( Fig. 1 **d** ). Preoperative staging via enhanced computed tomography for gastric cancer indicated a submucosal bulge in the duodenal bulb, accompanied by small surrounding lymph nodes ( Fig. 1 **e, f** ). The preoperative assessment raised a high suspicion of a mesenchymal tumor, leading to the proposal of endoscopic submucosal dissection (ESD) as a treatment.

**Fig. 2:**
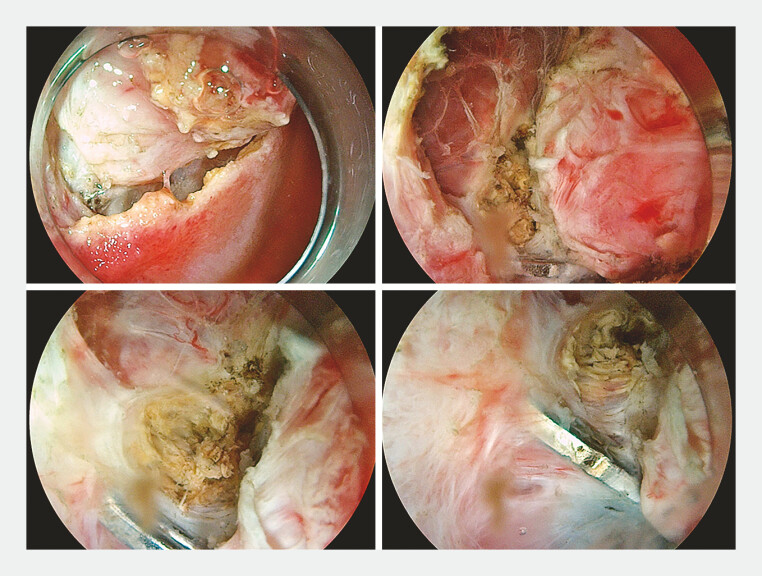
Metal clips were identified at the periphery of the lesion, which exhibited a close connection to the lesion.

**Fig. 3:**
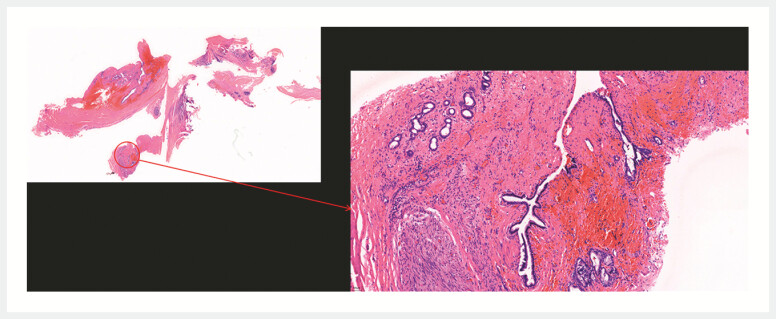
Postoperative pathological examination revealed a bile duct-derived glandular epithelium.

During the ESD procedure ( Video 1 ), the lesion was incised along its periphery and dissected layer by layer along the margins. A metal clip was identified at the central edge of the lesion, which was closely associated with the lesion and was considered a residual clip from a previous cholecystectomy ( Fig. 2 ). Given the proximity to the hepatic hilum, portal vein, and bile ducts and the associated high surgical risk, the procedure was halted at this stage. The patient was subsequently referred to the gastrointestinal surgery department for the laparoscopic resection of the duodenal mass on the same day. Postoperative pathological examination revealed fibrocystic wall-like tissue with interstitial collagenization and bile duct-derived adenoepithelium in some areas without malignant components, confirming the presence of a residual gallbladder ( Fig. 3 ).

A case involving a submucosal bulge in the duodenal bulb was ultimately confirmed to be a residual gallbladder.Video 1

The remnant gallbladder is described as the wider portion of the free end of the remnant choledochal duct, defined as a remnant duct greater than 1 cm in length 1 , and occurs after both open and laparoscopic cholecystectomy 2 . Therefore, endoscopists must consider the possibility of a remnant gallbladder in patients who have undergone cholecystectomy if a submucosal lesion is observed in the duodenal bulb.

Endoscopy_UCTN_Code_CCL_1AF_2AF
